# Digital Horizons: Enhancing Autism Support with Augmented Reality

**DOI:** 10.1007/s10803-024-06709-4

**Published:** 2025-02-28

**Authors:** Yiannis Koumpouros

**Affiliations:** Director of Digital Innovation in Public Health Research Lab (DigInHealth), Department of Public and Community Health, University of West Attica, Egaleo, Greece

**Keywords:** Autism, Augmented Reality, AR, ASD, Autism Spectrum Disorder, Disability, ADHD, Mixed Reality, Virtual Reality

## Abstract

**Purpose:**

This paper aims to comprehensively review the application of Augmented Reality interventions in supporting individuals diagnosed with Autism Spectrum Disorder. The main research question guiding this review is: What effects do AR interventions have on various aspects of functioning in individuals with ASD?

**Methods:**

A systematic review methodology was employed to analyze 49 articles published between 2013 and 2023. These articles were selected based on their relevance to AR interventions for individuals with ASD. The review examines the diverse technological landscape of AR interventions, the various platforms utilized, and the effectiveness of different AR techniques.

**Results:**

Findings reveal the prevalence of smartphones, tablets, smart glasses, and head-mounted displays as primary platforms for AR interventions, with positive outcomes reported across various domains including social interaction skills, communication abilities, and academic performance. Marker-based, superimposition-based, and projection-based AR techniques demonstrate potential in creating personalized and engaging experiences tailored to the unique needs of individuals with ASD.

**Conclusion:**

Despite progress in communication and social skills interventions, gaps remain in understanding and addressing attention-related issues and emotion recognition. The review underscores the need for more rigorous study designs and objective evaluation methods to ascertain the efficacy of AR interventions for individuals with ASD. Looking ahead, collaborative efforts between researchers, developers, practitioners, and individuals with ASD are crucial for advancing innovation, addressing limitations, and ensuring the meaningful integration of AR technology into interventions aimed at enhancing the quality of life for individuals on the autism spectrum. Further exploration and utilization of the latest advancements in artificial intelligence and affective computing are warranted to develop solutions that effectively address real-world challenges faced by individuals with autism.

**Supplementary Information:**

The online version contains supplementary material available at 10.1007/s10803-024-06709-4.

## Introduction

Autism Spectrum Disorder (ASD) is a multifaceted neurodevelopmental condition characterized by challenges in social communication, repetitive behaviors, and restricted interests (Jones & Klin, 2013a). These challenges significantly impact learning and daily functioning (American Psychiatric Association, 2013). The diverse and intricate nature of ASD necessitates innovative approaches to support individuals across various developmental domains. While traditional interventions such as behavioral therapies, speech therapy, and occupational therapy have been instrumental in supporting individuals with ASD (Reichow et al., 2012; Rogers & Vismara, 2008), the integration of technology offers novel opportunities for personalized and engaging interventions (Koumpouros & Kafazis, 2019).

Augmented Reality (AR) has emerged as a promising tool for enhancing educational and therapeutic experiences for individuals with ASD (Koumpouros, 2024). The application of AR in the context of autism has garnered attention due to its potential to offer interactive and immersive experiences that cater to the unique needs of individuals on the spectrum (Lahiri et al., 2013a). AR enhances users’ perception of the environment by overlaying digital elements (Milgram & Kishino, 1994), promoting interaction and learning.

Recent research underscores the potential of AR to improve social skills, communication, and learning in ASD. However, challenges such as accessibility, usability, and ethical considerations remain. Understanding the effects of AR interventions on individuals with ASD, as well as identifying best practices and guidelines for implementing AR in autism support, are critical areas for further research and development. This review aims to provide a comprehensive synthesis of the existing literature regarding the application of AR technology in the context of autism, shedding light on its applications, benefits, challenges, and future directions for research and practice. By mapping the breadth and depth of available research, this review seeks to identify the range of AR interventions, elucidate their methodologies, and critically evaluate their outcomes in supporting individuals with ASD. Furthermore, it aims to highlight gaps in current knowledge and delineate potential avenues for future research and development in this evolving field. With this in mind the primary research inquiry was, “*What evidence exists regarding the effects of AR interventions on enhancing various aspects of functioning in individuals with ASD?”* To address this question, a series of sub-questions were formulated to explore different facets:

R1. What types of AR interventions are available for individuals with ASD, and what specific areas do they target (e.g., social skills, communication, learning, behavior)?

R2. What methodological approaches are employed in studies assessing AR interventions for ASD (e.g., study design, participant characteristics, data collection methods, outcome measures)?

R3. What outcomes are reported from these interventions, and are there any factors that moderate or mediate their effectiveness (e.g., individual traits, features of intervention design)?

R4. What gaps exist in our understanding of the impact of AR interventions on individuals with ASD (e.g., insufficient research in certain domains, methodological constraints, factors affecting effectiveness)?

R5. What potential future research and development directions exist for AR interventions for ASD, taking into account ethical considerations and challenges in real-world implementation?

## Methods

### Research Objective and Scope

The primary aim of this scoping review is to systematically examine and map the existing literature from 2013 to 2023 on the utilization of AR interventions in individuals diagnosed with ASD and identify key themes and gaps. The review was conducted following the PRISMA (Preferred Reporting Items for Systematic Reviews and Meta-analyses) review protocol to ensure transparency and rigor in the review process (Kitchenham, 2004; Moher et al., 2009).

### Search Strategy

A comprehensive search strategy was devised to retrieve relevant articles from electronic databases including Science Direct, PubMed, MDPI, Google Scholar, Scopus, PsycINFO, Web of Science, ACM Digital Library, and IEEE Xplore for the period between 2013 and 2023 according to PICO (patient/population, intervention, comparison, and outcomes) (Stone, 2002). Keywords encompassing terms related to “autism,” “augmented reality,” “intervention,” and variations thereof were employed. Boolean operators were used to enhance search precision. The principal investigator, an IT expert in healthcare, supervised the search strategy. The initial pool of data was collected by an undergraduate student under the close supervision and guidance of an expert researcher in the field.

### Eligibility Criteria

Based on the PICO framework, inclusion criteria were: **(i) Population**: Individuals with Autism Spectrum Disorder across all ages and severities. **(ii) Intervention**: Use of AR targeting aspects like social communication, learning, behavior, education, and skill development. **(iii) Comparison**: No specific comparator due to the scoping nature of the review. **(iv) Outcome**: Improvements in social interaction, communication, behavior, education, etc.

Only full articles in English, including journal articles, conference papers, book chapters, and lecture notes, were included in the review. The inclusion of published conference papers and lecture notes is common in systematic reviews, as they often present significant findings before journal publication, reducing publication bias and offering a broader understanding of the research landscape. This approach is supported by prior studies (Hopewell et al., 2005; Paez, 2017) and systematic reviews by Koumpouros (2024) and Koumpouros and Kafazis (2019), validating the value of these sources for emerging research insights.

### Study Selection

The initial screening was conducted based on titles and abstracts against the predefined inclusion criteria. Full-text articles were then retrieved for further evaluation.

### Data Extraction

A standardized data extraction form was developed to extract relevant information from included studies. Key data points encompassed study characteristics, AR intervention details, participant details, outcome measures, and findings related to the impact of AR interventions on individuals with ASD. At the beginning of the data extraction process, a set of five research questions was identified to guide the analysis, as indicated in previous paragraphs (R1-R5).

### Synthesis of Results

A narrative synthesis approach was employed to systematically summarize and interpret findings from included studies. Themes and patterns emerging from the data were identified, and a descriptive overview of the scope and nature of AR interventions in the context of autism was developed (Tricco et al., 2018). The results of this review are reported in accordance with PRISMA guidelines, providing a transparent and comprehensive overview of the identified literature.

## Results

**Fig. 1:**
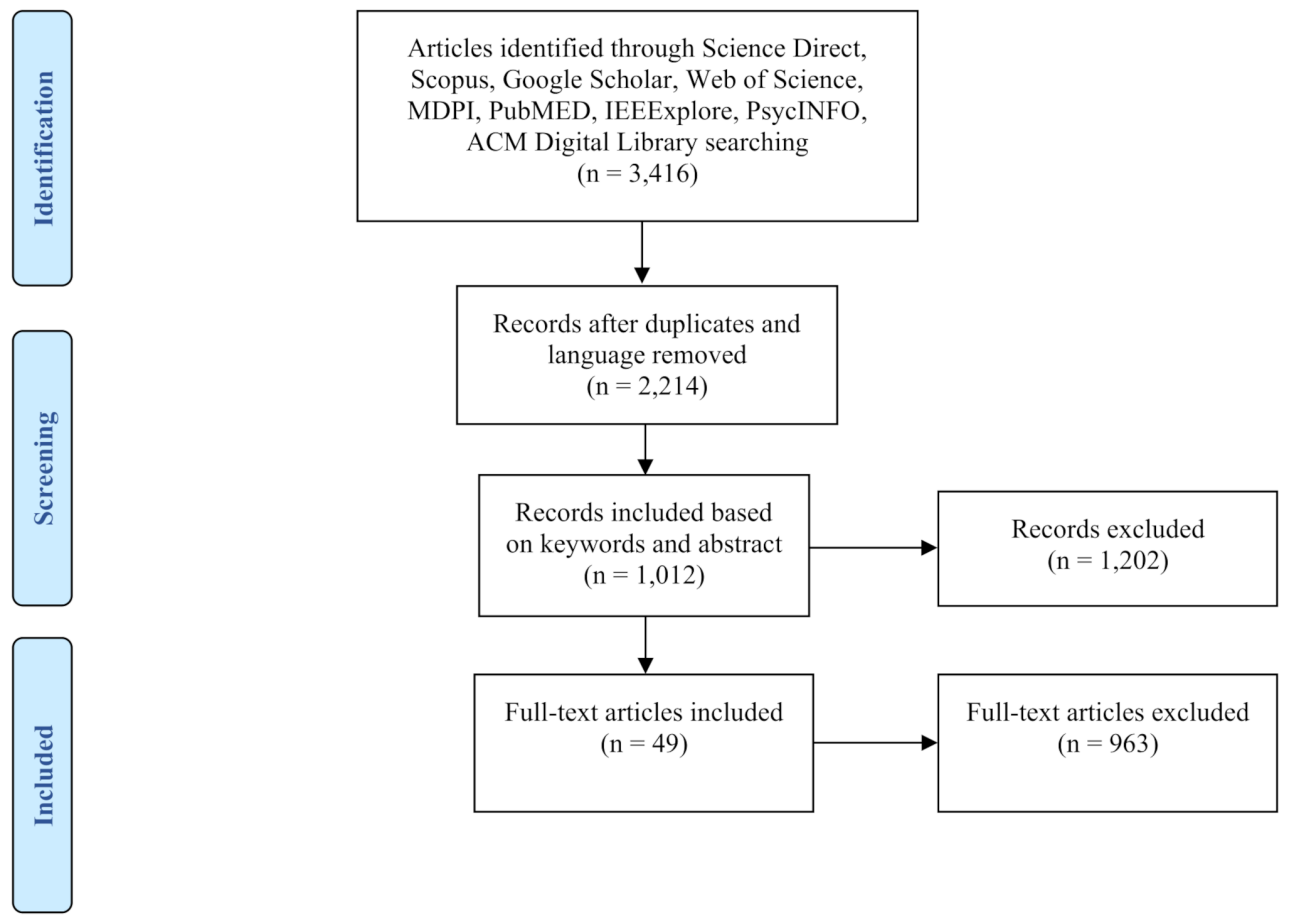
PRISMA flowchart

The initial search identified 3,625 articles. After removing duplicates and irrelevant studies, 49 articles met the inclusion criteria. These studies explored various AR applications, with most published in journals (53.1%), conferences (30.6%), or lecture notes (16.3%). The comprehensive selection process is delineated in Fig. 1, while the supplementary Fig. 2 presents the frequency of publications over time.

The results indicate that 53.1% of the examined articles were published in journals, 30.6% in conferences, and 16.3% in Lecture Notes. Published conferences and lecture notes are included to capture the breadth of research being conducted in this field, recognizing that innovative findings and preliminary studies are often presented in these formats before being published in peer-reviewed journals. Table 1 delineates the specific technological methods employed in each study, revealing that approximately half of the studies utilized marker-based techniques, 38.5% utilized superimposition-based methods, while 5.8% opted for a projection-based approach, and 1.9% utilized overlay techniques.

**Table 1:** Technological features

#	Paper	Developer tools	Operating system	Device used	AR type
1	Liu, R., Salisbury, J. P., Vahabzadeh, A., & Sahin, N. T. (2017). Feasibility of an Autism-Focused Augmented Reality Smartglasses System for Social Communication and Behavioral Coaching. Frontiers in Pediatrics, 5, 145. 10.3389/fped.2017.00145	BPS SDK	iOS, Android	Google Glass Explorer Edition v2	M
2	Nekar, D. M., Kang, H., Alao, H., & Yu, J. (2022). Feasibility of Using Multiplayer Game-Based Dual-Task Training with Augmented Reality and Personal Health Record on Social Skills and Cognitive Function in Children with Autism. Children, 9(9), 1398.	Kinect SDK	Windows	Kinect	S
3	Vahabzadeh, A., Keshav, N. U., Abdus-Sabur, R., Huey, K., Liu, R., & Sahin, N. T. (2018). Improved socio-emotional and behavioral functioning in students with autism following school-based smartglasses intervention: Multi-stage feasibility and controlled efficacy study. Behavioral Sciences, 8(10), 85.	Empowered Brain Technology Platform	iOS, Android, Glass OS	Empowered Brain smartglasses	S
4	Sahin, N. T., Keshav, N. U., Salisbury, J. P., & Vahabzadeh, A. (2018). Second version of google glass as a wearable socio-affective aid: Positive school desirability, high usability, and theoretical framework in a sample of children with autism. JMIR Human Factors, 5(1), e8785.	-	-	Glass Enterprise Edition	S
5	Krishnamurthy, D., Jaswal, V., Nazari, A., Shahidi, A., Subbaraman, P., & Wang, M. (2022, April). HoloType: Lived Experience Based Communication Training for Nonspeaking Autistic People. In CHI Conference on Human Factors in Computing Systems Extended Abstracts (pp. 1–6).	-	Windows	Hololens 2	S
6	Ahsen, T., Yu, C., O’Brien, A., Schlosser, R. W., Shane, H. C., Oesch-Emmel, D.,… & Dogar, F. (2022, October). Designing a Customizable Picture-Based Augmented Reality Application For Therapists and Educational Professionals Working in Autistic Contexts. In Proceedings of the 24th International ACM SIGACCESS Conference on Computers and Accessibility (pp. 1–16).	Unity, ARCore, ARKit XR	iOS, Android	smartphone	M
7	Hashim, H. U., Yunus, M. M., & Norman, H. (2022). ‘AReal-Vocab’: An Augmented Reality English Vocabulary Mobile Application to Cater to Mild Autism Children in Response towards Sustainable Education for Children with Disabilities. Sustainability, 14(8), 4831.	Unity	-	smartphone	M
8	Nekar, D. M., Lee, D. Y., Hong, J. H., Kim, J. S., Kim, S. G., Seo, Y. G., & Yu, J. H. (2022, October). Effects of Augmented Reality Game-Based Cognitive–Motor Training on Restricted and Repetitive Behaviors and Executive Function in Patients with Autism Spectrum Disorder. Healthcare, 10(10), 1981.	UINCARE	Windows	UINCARE-82B (PC, kinect), CoTras device	M
9	Wan, G., Deng, F., Jiang, Z., Song, S., Hu, D., Chen, L.,… & Zhang, J. (2022). FECTS: A Facial Emotion Cognition and Training System for Chinese Children with Autism Spectrum Disorder. Computational Intelligence and Neuroscience, 2022.	OpenFace SDK	Android	PAD with a training system, PC with an analysis system, mini Bluetooth speaker, D435 camera	S
10	Wedyan, M., Falah, J., Alturki, R., Giannopulu, I., Alfalah, S. F., Elshaweesh, O., & Al-Jumaily, A. (2021). Augmented reality for autistic children to enhance their understanding of facial expressions. Multimodal Technologies and Interaction, 5(8), 48.	Unity	Windows	Kinect	S
11	Lee (2021). Kinect-for-windows with augmented reality in an interactive roleplay system for children with an autism spectrum disorder. Interactive Learning Environments, 29(4), 688–704.	Unity	Windows	Kinect	S
12	Wang, C. P., Tsai, C. H., & Lee, Y. L. (2022). Requesting Help Module Interface Design on Key Partial Video with Action and Augmented Reality for Children with Autism Spectrum Disorder. Applied Sciences, 12(17), 8527.	Unity, Vuforia, HP Reveal, AR Kit, MAKAR	iOS, Android, Unity 3D	tablet	M
13	Xia, M., Chen, N., Tang, Y., & Zhu, Z. (2020, October). ParaShop: A Mobile AR App in Assisting People with ASD in Shopping. In 2020 IEEE MIT Undergraduate Research Technology Conference (URTC) (pp. 1–4). IEEE.	React Native, ViroReact, Node.js, PostgreSQL, Python Flask	iOS, Android	smartphone	M
14	Sun et al., (2019, B3October). Anon-Emoji: An optical see-Through augmented reality system for children with autism spectrum disorders to promote understanding of facial expressions and emotions. In 2019 IEEE International Symposium on Mixed and Augmented Reality Adjunct (ISMAR-Adjunct) (pp. 448–450). IEEE.	Unity, OpenCV	Magic Leap One Headset, OS version 0.95.2	Magic Leap One Headset	M
15	Tang, T. Y., Xu, J., & Winoto, P. (2019, July). An augmented reality-based word-learning mobile application for children with autism to support learning anywhere and anytime: object recognition based on deep learning. In International Conference on Human-Computer Interaction (pp. 182–192). Springer, Cham.	TensorFlow	iOS, Android	smartphone	M
16	Lee, I. J., Chen, C. H., Wang, C. P., & Chung, C. H. (2018). Augmented reality plus concept map technique to teach children with ASD to use social cues when meeting and greeting. The Asia-Pacific Education Researcher, 27(3), 227–243.	Vuforia	-	tablet	M
17	Vahabzadeh, A., Keshav, N. U., Salisbury, J. P., & Sahin, N. T. (2018). Improvement of attention-deficit/hyperactivity disorder symptoms in school-aged children, adolescents, and young adults with autism via a digital smartglasses-based socioemotional coaching aid: short-term, uncontrolled pilot study. JMIR Mental Health, 5(2), e9631.	Empowered Brain Technology Platform	iOS, Android, Glass OS	Empowered Brain system, smartglasses	S
18	Keshav, N. U., Vahabzadeh, A., Abdus-Sabur, R., Huey, K., Salisbury, J. P., Liu, R., & Sahin, N. (2018). Longitudinal socio-emotional learning intervention for autism via smartglasses: Qualitative school teacher descriptions of practicality, usability, and efficacy in general and special education classroom settings. Education Sciences, 8(3), 107.	Empowered Brain Technology Platform	iOS, Android, Glass OS	Empowered Brain system, Google glasses	S
19	Asif, M. A., Al Wadhahi, F., Rehman, M. H., Kalban, I. A., & Achuthan, G. (2020). Intelligent educational system for autistic children using augmented reality and machine learning. In Innovative Data Communication Technologies and Application: ICIDCA 2019 (pp. 524–534). Springer International Publishing.	OpenCV, Kinect SDK	Windows	Kinect, short throw projector, webcam	M, P
20	Chung, C. H., & Chen, C. H. (2017). Augmented reality based social stories training system for promoting the social skills of children with autism. In Advances in Ergonomics Modeling, Usability & Special Populations: Proceedings of the AHFE 2016 International Conference on Ergonomics Modeling, Usability & Special Populations, July 27–31, 2016, Walt Disney World^®^, Florida, USA (pp. 495–505). Springer international publishing.	-	-	laptop	M
21	Sahin, N. T., Abdus-Sabur, R., Keshav, N. U., Liu, R., Salisbury, J. P., & Vahabzadeh, A. (2018, September). Case study of a digital augmented reality intervention for autism in school classrooms: Associated with improved social communication, cognition, and motivation via educator and parent assessment. In Frontiers in education (Vol. 3, p. 57). Frontiers Media SA.	Empowered Brain Technology Platform	iOS, Android, Glass OS	Empowered Brain system, Google glasses	S
22	Lorenzo et al., (2019). Preliminary study of augmented reality as an instrument for improvement of social skills in children with autism spectrum disorder. Education and Information Technologies, 24, 181–204.	Quiver Vision SDK	Android	smartphone	S
23	Huang, Y. C., & Lee, I. J. (2020). Using augmented reality and concept mapping to improve ability to master social relationships and social reciprocity for children with autism Spectrum disorder. In Universal Access in Human-Computer Interaction. Applications and Practice: 14th International Conference, UAHCI 2020, Held as Part of the 22nd HCI International Conference, HCII 2020, Copenhagen, Denmark, July 19–24, 2020, Proceedings, Part II 22 (pp. 19–37). Springer International Publishing.	Unity, 3DSmax, After Effects	MacOS, Windows	laptop	M
24	Kurniawan (2018). The improvement of autism spectrum disorders on children communication ability with PECS method Multimedia Augmented Reality-Based. In Journal of Physics: Conference Series (Vol. 947, No. 1, p. 012009). IOP Publishing.	-	Android	smartphone, tablet	M
25	Zheng, C., Zhang, C., Li, X., Li, B., Zhang, F., Liu, X.,… & Ying, F. (2017, October). An EEG-based adaptive training system for ASD children. In Adjunct Proceedings of the 30th Annual ACM Symposium on User Interface Software and Technology (pp. 197–199).	Face + + SDK	MacOS, Windows, Android, iOS	EEG helmet, TGAM chip module, laptop	M
26	Wang, W. Z., & Lee, I. J. (2020). Social Intervention Strategy of Augmented Reality Combined with Theater-Based Games to Improve the Performance of Autistic Children in Symbolic Play and Social Skills. In HCI International 2020–Late Breaking Papers: Universal Access and Inclusive Design: 22nd HCI International Conference, HCII 2020, Copenhagen, Denmark, July 19–24, 2020, Proceedings 22 (pp. 401–411). Springer International Publishing.	Unity, Vuforia, Maya	iOS, Android	tablet	M
27	Keshav, N. U., Salisbury, J. P., Vahabzadeh, A., & Sahin, N. T. (2017). Social communication coaching smartglasses: Well tolerated in a diverse sample of children and adults with autism. JMIR mHealth and uHealth, 5(9), e8534.	Google Glass Explorer Edition and Glass Enterprise Edition	iOS, Android, Glass OS	Google Glass Explorer Edition and Glass Enterprise Edition, smartglasses	S
28	Chen, C. H., Lee, I. J., & Lin, L. Y. (2016). Augmented reality-based video-modeling storybook of nonverbal facial cues for children with autism spectrum disorder to improve their perceptions and judgments of facial expressions and emotions. Computers in Human Behavior, 55, 477–485.	ARVMS learning system	Windows	tablet PC	O
29	Pérez-Fuster et al., (2022). Enhancing joint attention skills in children on the autism spectrum through an augmented reality technology-mediated intervention. Children, 9(2), 258.	Kinect SDK, Pictogram Room	Windows	Kinect, Projector, Interactive Digital Whiteboard, PC, video cameras	S
30	Chen, C. H., Lee, I. J., & Lin, L. Y. (2015). Augmented reality-based self-facial modeling to promote the emotional expression and social skills of adolescents with autism spectrum disorders. Research in Developmental Disabilities, 36, 396–403.	Unity, Vuforia, 3Ds Max, Qualcom AR	Windows	PC, Monitor, Web camera	S
31	Zheng, C., Zhang, C., Li, X., Liu, X., Tang, C., Wang, G.,… & Ying, F. (2017). Toon-chat: a cartoon-masked chat system for children with autism. In ACM SIGGRAPH 2017 Posters (pp. 1–2).	Face + + SDK	MacOS, Windows, Android, iOS	laptop, monitor	S
32	Mahariba, A. J., Mishra, S., & Jain, D. (2016). Face morphing and substitution for aid of autistic children using augmented reality. Indian J. Sci. Technol., 9(37).	OpenCV, Unity	MacOS, Windows, Android, iOS	PC, monitor	S
33	McMahon, D. D., Cihak, D. F., Wright, R. E., & Bell, S. M. (2016). Augmented reality for teaching science vocabulary to postsecondary education students with intellectual disabilities and autism. Journal of Research on Technology in Education, 48(1), 38–56.	Aurasma	iOS	iPad	M
34	Lakshmiprabha, N. S., Santos, A., Mladenov, D., & Beltramello, O. (2014, September). [Poster] An augmented and virtual reality system for training autistic children. In 2014 IEEE International Symposium on Mixed and Augmented Reality (ISMAR) (pp. 277–278). IEEE.	OpenCV	MacOS, Windows, Android, iOS	projector, camera	*P*
35	Escobedo and Tentori (2014). Mobile augmented reality to support teachers of children with autism. In Ubiquitous Computing and Ambient Intelligence. Personalisation and User Adapted Services: 8th International Conference, UCAmI 2014, Belfast, UK, December 2–5, 2014. Proceedings 8 (pp. 60–67). Springer International Publishing.	MOBIS	-	smartphone	*P*
36	Vullamparthi, A. J., Nelaturu, S. C. B., Mallaya, D. D., & Chandrasekhar, S. (2013, December). Assistive learning for children with autism using augmented reality. In 2013 IEEE Fifth International Conference on Technology for Education (t4e 2013) (pp. 43–46). IEEE.	Eclipse 4.0 Indigo, PHP, Java	Android, Windows	tablet, smartphone	M
37	Alkadhi et al., (2020). Co-design of Augmented Reality Storybooks for Children with Autism Spectrum Disorder. In HCI International 2020–Late Breaking Papers: Universal Access and Inclusive Design: 22nd HCI International Conference, HCII 2020, Copenhagen, Denmark, July 19–24, 2020, Proceedings 22 (pp. 3–13). Springer International Publishing.	-	-	smartphone	M
38	Singh, K., Shrivastava, A., Achary, K., Dey, A., & Sharma, O. (2019, November). Augmented reality-based procedural task training application for less privileged children and autistic individuals. In The 17th International Conference on Virtual-Reality Continuum and its Applications in Industry (pp. 1–10).	Unity	MacOS, Windows	PC	M
39	Kung-Teck, W., Hanafi, H. F., Abdullah, N., Noh, N. M., & Hamzah, M. (2019). A prototype of augmented reality animation (ara) e-courseware: An assistive technology to assist autism spectrum disorders (asd) students master in basic living skills. International Journal of Innovative Technology and Exploring Engineering, 9(1), 3487–3492.	-	-	tablet, smartphone	M
40	Premarathne, H. (2022, March). [DC] A Mobile Intervention to Promote Social Skills in Children with Autism Spectrum Disorder Using AR Face Masks. In 2022 IEEE Conference on Virtual Reality and 3D User Interfaces Abstracts and Workshops (VRW) (pp. 942–943). IEEE.	Unity	iOS, Android	smartphone	S
41	Bauer, V., Bouchara, T., & Bourdot, P. (2021, October). Designing an extended reality application to expand clinic-based sensory strategies for autistic children requiring substantial support: participation of practitioners. In 2021 IEEE International Symposium on Mixed and Augmented Reality Adjunct (ISMAR-Adjunct) (pp. 254–259). IEEE.	Unity, Steam VR SDK, ZED SDK	Windows	HTC Vive Pro, Zed mini camera	S
42	Amado, M. L., Ruiz, L. C., & Andrade-Arenas, L. (2021). Prototype of an augmented reality application for cognitive improvement in children with autism using the DesingScrum methodology. Advances in Science, Technology and Engineering Systems, 6(1), 587–596.	Balsamiq Mockups 3, Tinkercad, App Augmented Class, Unity, Vuforia	iOS, Android	smartphone	M
43	Khowaja, K., Al-Thani, D., Abdelaal, Y., Hassan, A. O., Mou, Y. A., & Hijab, M. H. (2021, July). Towards the Mixed-Reality Platform for the Learning of Children with Autism Spectrum Disorder (ASD): A Case Study in Qatar. In HCI in Games: Serious and Immersive Games: Third International Conference, HCI-Games 2021, Held as Part of the 23rd HCI International Conference, HCII 2021, Virtual Event, July 24–29, 2021, Proceedings, Part II (pp. 329–344). Cham: Springer International Publishing.	-	-	smartphone, tablet	M
44	Bouaziz et al., (2021). Enhancing Daily Life Skills Learning for Children with ASD Through Augmented Reality. In Innovative Systems for Intelligent Health Informatics: Data Science, Health Informatics, Intelligent Systems, Smart Computing (pp. 1164–1173). Cham: Springer International Publishing.	Unity, Blender	Android	smartphone	M
45	Iftene and Trandabăț (2018). Enhancing the attractiveness of learning through augmented reality. Procedia Computer Science, 126, 166–175.	Artoolkit framework, Metaio framework, Unity, Vuforia	Android	smartphone, tablet	M
46	Tang, T. Y., Xu, J., & Winoto, P. (2019, March). Automatic object recognition in a light-weight augmented reality-based vocabulary learning application for children with autism. In Proceedings of the 2019 3rd international conference on innovation in artificial intelligence (pp. 65–68).	Google TensorFlow platform	iOS, Android	smartphone	S
47	Cunha, P., Brandão, J., Vasconcelos, J., Soares, F., & Carvalho, V. (2016, February). Augmented reality for cognitive and social skills improvement in children with ASD. In 2016 13th International Conference on Remote Engineering and Virtual Instrumentation (REV) (pp. 334–335). IEEE.	-	-	smartphone, tablet, laptop	M
48	Li et al., (2023). FaceMe: An agent-based social game using augmented reality for the emotional development of children with autism spectrum disorder. International Journal of Human-Computer Studies, 175, 103,032.	Unity, Vuforia, FaceMe system	Android	laptop	S
49	Lian, X., Sunar, M. S., Lian, Q., & Mokhtar, M. K. (2023). Evaluating user interface of a mobile augmented reality coloring application for children with autism: An eye-tracking investigation. International Journal of Human-Computer Studies, 103,085.	Vuforia, OpenCV	Android	smartphone	M

Regarding devices, smartphones (36.7%) and tablets (24.5%) were most commonly used, with smart glasses or head-mounted displays employed in 18.4% of studies. One study (2%) utilized an interactive board, and another employed an EEG helmet (2%). Operating systems varied, with Android (53.1%) and iOS (38.8%) being the most prevalent. Windows was utilized in 34.7% of cases, MacOS in 12.2%, and the operating system was not reported in 18.4% of studies (*n* = 9). Unity emerged as the predominant tool, utilized in 22.4% of articles, followed by Vuforia (10.2%) and the Empowered Brain Technology Platform (8.2%). Kinect SDK, OpenFace SDK, Face + + SDK, and Artoolkit framework collectively accounting for 14.3% of the articles. Other tools and platforms, including React Native, Node.js, Python Flask, OpenCV, TensorFlow, Maya, Aurasma, MOBIS, Eclipse, PHP, Java, Steam VR SDK, ZED SDK, Balsamiq Mockups 3, and Tinkercad, were individually mentioned in 16.3% of the articles. Some articles (14.3%) did not specify any particular developer tool.

The studies covered a diverse range of fields, with communication and social skills being the most common focus area (53.1%), followed by cognitive function, attention-related issues, and sciences (biology, math, geography) (8.2% each). Additionally, therapy (4.1%), emotion recognition (2%), rehabilitation training (2%), ADHD-related symptoms (2%) were targeted. Vocabulary learning (10.2%), education (10.2%), reading (2%), and daily living skills (6.1%) are also targeted. Finally, technology use is explored in one study (2%). A supplementary Fig. 3 provides a visual breakdown of the focus areas of AR interventions to complement the findings presented.

The researchers assessed the final application’s effectiveness through a variety of methods. The majority (76.9%) relied on subjective evaluation, gathering user opinions and experiences. Conversely, 26.9% employed objective assessment techniques, using measurable data like task completion rates or system logs to evaluate the application’s performance. Notably, 9.6% of the studies did not include any evaluation of the proposed solution.

Within the subjective evaluation methods, observation techniques were the most common (49%, *n* = 24). Researchers directly observed users interacting with the application to understand their behavior and identify any usability issues. Participant interviews (46.9%, *n* = 23) were another popular approach, allowing users to share their thoughts and feelings about the application in their own words. Additionally, 26.5% of the studies (*n* = 13) utilized custom-made questionnaires designed specifically for their project to gather user feedback. In contrast, only 6 studies (12.2% total) incorporated already established, valid and reliable questionnaires that have been proven effective in measuring user experience or application performance.

More specifically, two studies (4.1%) used the Questionnaire for User Interaction Satisfaction (QUIS) to evaluate user satisfaction with the technology. Another study utilized the Stroop Color and Word Test (SCWT), a task designed to assess cognitive control and processing speed. Additionally, the Social Responsiveness Scale 2 (SRS-2) was used in one study to measure social behavior and communication, while another study applied the Early Social Communication Scales (ESCS) to assess early social communication skills. It is important to note that these measures and tasks assess distinct constructs, which must be considered when interpreting the results.

Of the 49 articles analyzed, only one (2.0%) included a control group in its research. Seven studies (14.3%) concentrated on students, while eight (16.3%) addressed educators or teachers. Two studies (4.1%) were geared towards therapists, and two (4.1%) towards parents. Seven studies (14.3%) involved participants with typical development to establish baseline data for comparison with ASD populations, one (2.0%) involved psychologists, and three (6.1%) targeted other specific end user groups. Additionally, eight studies (16.3%) that target ASD population did not provide details of the type of end users involved (e.g., age). Despite this limitation, all studies were ultimately included in the review due to their focus on the ASD population and the use of AR.

A comprehensive overview of the findings, constraints, and future directions of the examined articles is provided in suplementary Table 2. The effectiveness of AR interventions varied across studies, with 77.6% (38 out of 49 studies) reporting positive outcomes, including significant improvements in targeted skills, such as social communication, verbal and nonverbal communication, and repetitive behaviors. However, 4.1% (2 studies) indicated no statistically significant differences between AR and non-AR intervention groups, highlighting variability in outcomes. Additionally, 18.4% (9 studies) focused on typical development or did not specify end users, providing limited insights into the specific needs and responses of individuals with ASD. This highlights a significant gap in the literature, emphasizing the need for more targeted research that clearly identifies and addresses the unique challenges faced by individuals with ASD.

Supplementary Table 3 presents the categorization and examination of the reviewed articles based on Koumpouros’s (2024) taxonomy, facilitating the synthesis of results and the identification of research trends and deficiencies. This taxonomy offers a systematic framework that supports educators and researchers in organizing, grouping, and understanding the various facets of integrating AR technology into educational settings.

We critically evaluated the strengths and limitations of each study, discussing the methodological rigor and implications of the findings. This involved a detailed examination of the study designs, sample sizes, intervention protocols, and outcome measures. Methodological approaches were scrutinized to identify potential biases and limitations that could affect the reliability and validity of the results. For instance, we noted that many studies had small sample sizes and lacked control groups, limiting the generalizability of their findings. Additionally, we highlighted the variability in outcome measures used across studies, complicating the comparison of results and the synthesis of evidence.

The paper provides a balanced view of the effectiveness of AR interventions, acknowledging both the potential benefits and the current gaps in the evidence. While the studies reviewed show promising results in certain areas, the overall quality of evidence remains low due to methodological limitations. We emphasized the need for larger, well-designed studies with standardized outcome measures to establish the efficacy of AR interventions. We also discussed the potential of AR technology to create engaging and personalized learning experiences for individuals with ASD, while cautioning against over-reliance on these tools without sufficient evidence of their effectiveness. Our balanced perspective ensures that readers are aware of both the potential benefits and the limitations of AR interventions, guiding future research and practice in this field. A supplementary summary Table 4 provides a clear and concise snapshot of the key findings.

## Discussion

This review highlights AR’s transformative potential in ASD interventions, addressing critical domains such as communication, education, and therapy. The examination of 49 articles published between 2013 and 2023 revealed a diverse landscape of research, showcasing the multifaceted applications of AR in addressing the needs of individuals with ASD. Guided by the overarching question “What effects do AR interventions have on various aspects of functioning in individuals with ASD?“, five sub-questions explored specific facets of this topic.

### Technological Landscape Reflects Diverse Needs

The findings from the review highlight the diverse technological landscape of AR interventions for individuals with ASD. While a definitive association between specific technologies and outcomes is challenging due to study design variations, some trends and potential insights emerge from the provided data. Concerning the device usage, several studies (Liu et al., 2017; Ahsen et al., 2022; Hashim et al., 2022; Xia et al., 2020; Tang et al., 2019; Sahin et al., 2018; Wang & Lee, 2020; Escobedo & Tentori, 2014; Vullamparthi et al., 2013; Kung-Teck et al., 2019; Bauer et al., 2021; Khowaja et al., 2021; Iftene & Trandabăț, 2018; Lian et al., 2023) primarily employed smartphones or tablets for AR interventions. Positive outcomes encompassed improvements in social interaction skills, communication abilities, shopping skills, and academic performance.

AR technology shows significant potential across diverse applications for individuals with ASD, including cognitive rehabilitation, therapy, emotion recognition, and daily living skills. In educational contexts, AR was utilized to support vocabulary learning, reading, and science education, further demonstrating its broad applicability. Notably, the exploration of AR in fostering technology literacy, though still emerging, highlights the evolving potential of this technology in preparing individuals with ASD for increasingly digitalized environments. These findings emphasize the versatility of AR interventions, supporting both therapeutic and educational outcomes while opening new avenues for future research.

Additionally, smartphones were found to be effective in reducing teachers’ workload and enhancing multitasking abilities. Studies utilizing smart glasses or head-mounted displays (Vahabzadeh et al., 2018a, b; Sahin et al., 2018; Keshav et al., 2017) reported positive outcomes such as reduction in irritability, hyperactivity, and social withdrawal, as well as improvements in social communication, cognition, and motivation. Interventions utilizing Kinect and projectors (Nekar et al., 2022; Wan et al., 2022; Wedyan et al., 2021a, b; Lee, 2021; Asif et al., 2020; Pérez-Fuster et al., 2022; Lakshmiprabha et al., 2014) demonstrated improvements in various areas such as social awareness, cognition, communication, emotional recognition, and response to joint attention skills. AR interventions conducted on laptops or PCs (Chung & Cheng, 2017; Huang & Lee, 2020; Zheng et al., 2017; Mahariba et al., 2016; Vullamparthi et al., 2013; Li et al., 2023) led to improvements in learning effectiveness, social behaviors, concentration, and facial expression recognition. One study (Zheng et al., 2017) utilized an EEG helmet and reported encouraging results in understanding ASD children better. This system provided real-time EEG feedback to assess and adapt training sessions tailored to the individual needs of children with ASD. The results indicated that the adaptive system could improve engagement and focus during training sessions, helping children to better regulate their behaviors and responses. Additionally, the system showed potential for identifying patterns in neural activity associated with specific ASD-related challenges, offering valuable insights for developing personalized intervention strategies.

In line with the findings of Koumpouros (2024), smartphones and tablets dominated the platform choices (61.2%), underscoring their accessibility and diverse functionalities. The shift towards mobile augmented reality has been significant, with mobile devices becoming more powerful and affordable, enabling a wide range of AR applications. With the proliferation of smartphones equipped with advanced AR capabilities, such as depth-sensing cameras and powerful processors, mobile AR applications are increasingly being leveraged to enhance the social and cognitive skills of individuals on the autism spectrum. By overlaying virtual elements onto the real world, these applications provide personalized and interactive experiences tailored to the unique needs of individuals with ASD, facilitating learning, communication, and social interactions. For example, AR-based social skills training apps offer immersive scenarios that help individuals with autism navigate social cues and practice social interactions in controlled environments. Moreover, mobile AR games designed specifically for individuals with ASD promote engagement and sensory integration while fostering creativity and problem-solving skills. Recent research (Berenguer et al., 2020; Lian & Sunar, 2021) underscores the transformative potential of mobile AR in supporting individuals with autism, highlighting its ability to empower and enrich their lives through innovative and accessible interventions. The future of mobile AR for autism holds great promise in providing innovative solutions to support individuals with ASD in various aspects of their lives, promoting independence, communication, and social integration. The future of mobile augmented reality is closely intertwined with advancements in artificial intelligence and emotional computing, which enhances AR experiences.

The notable prevalence of smart glasses and head-mounted displays (18.4%) within the scope of this review corresponds to the timeframe under consideration, reflecting a growing interest in immersive experiences that could potentially benefit interventions targeting social skills and emotional recognition, as indicated in the sub-questions (R1). These wearable devices present a hands-free and immersive medium for delivering customized interventions and support tailored to the specific requirements of individuals on the autism spectrum. With ongoing technological advancements, including lightweight designs, high-resolution displays, and sophisticated sensors, head-mounted displays (HMDs) and smart glasses are increasingly becoming more accessible and practical for individuals with ASD. Particularly noteworthy is the debut of Apple’s latest wearable device, the Vision Pro, which has garnered attention for its advanced features aimed at enhancing accessibility and catering to diverse user needs, including those pertinent to autism. The Vision Pro integrates cutting-edge functionalities such as augmented reality overlays, advanced facial recognition, and real-time environmental awareness, allowing users to seamlessly integrate digital information with their surroundings (www.apple.com/apple-vision-pro/). However, the high cost of the Vision Pro presents a significant barrier to accessibility, especially when compared to more affordable alternatives such as the Oculus Quest. While the Vision Pro integrates AR functionalities, its focus on mixed reality (MR) and VR applications distinguishes it from more AR-centric devices. Additionally, other innovative products such as Microsoft HoloLens 2 and Google Glass Enterprise Edition 2 offer similar capabilities, presenting opportunities for individuals with autism to enhance social communication, sensory integration, and daily living skills through immersive and interactive experiences. Recent studies (Skjoldborg et al., 2022) have underscored the potential advantages of employing HMDs and smart glasses in the autism community, highlighting their role in fostering independence, engagement, and overall quality of life. Nonetheless, the utilization of HMDs and smart glasses is still in its nascent stage, and researchers are poised to explore the possibilities presented by new and exciting products, including cost-effective alternatives that cater specifically to the needs of individuals with ASD. The affordability of these products inevitably influences their broader adoption (Koumpouros, 2024). Furthermore, the range of other devices utilized, including interactive boards and EEG helmets, demonstrates the exploration of various technological avenues beyond traditional platforms, in line with recommendations to leverage the distinct strengths of different technologies (Koumpouros & Kafazis, 2019). A key point to consider in any application and research is, of course, the potential for autistic people to be hypersensitive to any wearable device.

The prevalence of marker-based techniques, superimposition-based methods, and projection-based approaches underscores the adaptability of AR technology to cater to various needs within the ASD community. Many studies (Liu et al., 2017; Ahsen et al., 2022; Hashim et al., 2022; Nekar et al., 2022; Wan et al., 2022; Wang et al., 2022; Lee et al., 2018; Asif et al., 2020; Chung & Chen, 2017; Sahin et al., 2018; Huang & Lee, 2020; Kurniawan, 2018; Zheng et al., 2017; Wang & Lee, 2020; Keshav et al., 2017; Zheng et al., 2017; Vullamparthi et al., 2013; Alkadhi et al., 2020; Singh et al., 2019; Kung-Teck et al., 2019; Khowaja et al., 2021; Iftene & Trandabăț, 2018; Tang et al., 2019; Cunha et al., 2016; Lian et al., 2023) employed marker-based AR techniques. For instance marker-based AR was used to enhance social communication skills through interactive social scenarios. Superimposition-based AR helped reduce sensory processing issues by overlaying calming visuals onto the environment. Projection-based AR was used in the study by Liu et al. (2017) to support academic skills through interactive educational games projected onto physical surfaces. Positive outcomes included improvements in social skills, communication, learning, attention, engagement, and academic performance. However, some studies (Singh et al., 2019; Lian et al., 2023) reported usability challenges for individuals with ASD when using marker-based AR. AR interventions utilizing superimposition-based methods (Nekar et al., 2022; Vahabzadeh et al., 2018a, b; Sahin et al., 2018; Krishnamurthy et al., 2022; Vahabzadeh et al., 2018a, b; Keshav et al., 2017, 2018; Lorenzo et al., 2019; Pérez-Fuster et al., 2022; Lakshmiprabha et al., 2014; Bauer et al., 2021; Amado et al., 2021; Khowaja et al., 2021; Iftene & Trandabăț, 2018; Tang et al., 2019) demonstrated positive outcomes such as reduction in ADHD-related symptoms, improvements in social communication, cognition, and motivation, as well as high tolerability and usability. Studies employing projection-based AR (Wan et al., 2022; Wedyan et al., 2021a, b; Asif et al., 2020; Pérez-Fuster et al., 2022; Lakshmiprabha et al., 2014) reported improvements in facial expression recognition, social interactions, talking, and emotional recognition. One study (Chen et al., 2016) utilizing overlay-based AR reported improvement in emotional recognition and social skills. Based on the results, marker-based techniques, which involve the use of physical markers or objects to trigger AR content, have been instrumental in creating interactive and engaging experiences for individuals with ASD, facilitating learning and communication in a personalized manner. Superimposition-based methods, where digital information is overlaid onto the real world, offer opportunities for individuals with ASD to enhance their sensory experiences and improve spatial awareness. Projection-based approaches, which project AR content onto physical surfaces, have shown promise in supporting social skills development and promoting interactive play among individuals with ASD. These varied approaches underscore the potential of AR technology to address a wide range of needs within the ASD community, providing tailored solutions for communication, education, and social interaction. Our study is in line with the research conducted by Bouaziz (2020) and Khowaja et al. (2020) highlighting the benefits of marker-based AR in improving social interaction skills and reducing social anxiety among individuals with ASD. Similarly, Porayska-Pomsta et al. (2012) demonstrated the efficacy of superimposition-based AR methods in enhancing communication abilities and promoting social engagement in ASD individuals. Additionally, recent findings from Wedyan et al. (2021a, b) showcased the utility of projection-based AR approaches in facilitating sensory integration and improving attentional focus in individuals with ASD. These studies collectively emphasize the versatility of AR technologies in addressing a wide range of needs within the ASD population, providing promising avenues for personalized interventions and support strategies. Another interesting finding is that iOS (38.8%) has gained more interest in the studied articles, indicating the trend for cross-platform applications. This aligns with the recent findings of Koumpouros (2024) suggesting the development of apps for both operating systems. Additionally, the identification of Unity as the predominant development tool underscores the importance of user-friendly software environments in facilitating the creation and implementation of AR interventions. The necessity for user-friendly software to develop AR applications is crucial, particularly for individuals such as teachers and therapists who may lack programming expertise but play vital roles in supporting individuals with ASD. Koumpouros (2024), Zhang et al. (2022), and Dechsling et al. (2022) collectively emphasize the critical role of user-friendly AR software in democratizing the development process and empowering educators and therapists to harness AR technology effectively in supporting individuals with ASD. Our findings suggest that different technologies used in AR interventions are associated with diverse outcomes in individuals with ASD. While smartphones and tablets were effective in improving various skills and reducing teachers’ workload, smart glasses and head-mounted displays showed promise in reducing symptoms and enhancing social communication and cognition. Additionally, marker-based AR techniques were widely used but posed usability challenges for some individuals with ASD, whereas superimposition-based methods demonstrated high tolerability and usability. Overall, the choice of technology may influence the effectiveness and usability of AR interventions for individuals with ASD, emphasizing the importance of considering specific needs and preferences when designing and implementing such interventions.

### Addressing Key Areas, but Gaps Remain

The review highlights AR interventions’ focus on communication and social skills, essential for the well-being of individuals with ASD. It also reflects a holistic approach by addressing cognitive function, special education, and therapy to meet diverse needs. We also identified evidence trends across the studies, including common outcomes and intervention strategies, while being mindful of the limitations inherent in the current body of research. Most studies focused on improving social interaction, communication skills, and academic performance in individuals with ASD. However, the diversity in intervention strategies and outcome measures across studies makes it challenging to draw definitive conclusions. Commonalities and differences in findings were discussed to provide a comprehensive overview of the current evidence. For example, while some studies reported significant improvements in social skills, others found no substantial effects, highlighting the need for further research with standardized methodologies. Despite this progress, significant gaps remain in understanding and applying AR interventions for individuals with ASD. The limited representation of attention-related issues, emotion recognition, and rehabilitation training in the literature highlights the need for further research in these areas. Advancements in artificial intelligence and affective computing hold considerable promise for research in emotion recognition (Shu et al., 2018). Additionally, leveraging deep learning techniques in conjunction with EEG signals presents novel opportunities in this domain (Wang et al., 2023; Wu et al., 2024). Thus, it is imperative for researchers to focus on exploring these avenues further. Additionally, the relatively low prevalence of interventions addressing vocabulary learning, daily living skills, ADHD-related symptoms, science vocabulary, and education points to areas where AR technology could be further leveraged to support individuals with ASD. The emphasis on communication and social skills (53.1%) aligns with previous findings underscoring AR’s efficacy in these areas (Yuan & Ip, 2018), while addressing cognitive function, special education, and therapy (8.2% each) highlights the versatility of AR interventions in addressing diverse challenges, consistent with initial hypotheses (R1). Targeting attention-related issues, emotion recognition, and rehabilitation training (2% each) suggests potential benefits for psychological and cognitive rehabilitation, an area warranting further exploration (Bakir et al., 2023). The inclusion of studies addressing vocabulary learning, daily living skills, ADHD-related symptoms, and educational settings (2% each) emphasizes the broad applicability of AR across diverse contexts, as explored in R1. Notably, exploring technology use itself (2%) reflects an emerging interest in utilizing AR to enhance technology literacy in individuals with ASD, potentially opening doors to new learning opportunities. However, the limited focus on specific end-user groups (other than students and educators) indicates a gap in addressing the diverse needs of different populations within the ASD spectrum, as identified in R4.

### Evaluation Methods - Strengths and Opportunities

In the examined articles, most studies targeted children, with varying age ranges and both typical development and ASD diagnoses. Only a limited number of studies targeted students and the final applications were used in school settings for academics and social skills (Vahabzadeh et al., 2018a, b; Keshav et al., 2018; Kung-Teck et al., 2019). Limited studies focused on adults with ASD (Xia et al., 2020; McMahon et al., 2016), while educators and therapists were involved in using or evaluating interventions (Ahsen et al., 2022; Escobedo & Tentori, 2014; Iftene & Trandabăț, 2018) in three studies. Another finding, was the small groups (2–10) of end users involved in the evaluation phase. Larger groups (12–30) were used in studies focusing on feasibility and broader applicability. There was no clear association between number of users and specific outcomes. The review underscores the diverse array of evaluation techniques utilized to gauge the effectiveness of AR interventions for individuals with ASD. The predominance of subjective evaluations (76.9%) reflects the inherent difficulties in implementing standardized assessments within dynamic AR environments (Koumpouros, 2024). Inherent difficulties in using standardized assessments in AR environments include variability in user interactions, differences in AR hardware and software, and the dynamic nature of AR experiences. These challenges can be addressed by developing standardized protocols for AR evaluations, creating adaptable AR content that can be used across different platforms, and conducting longitudinal studies to assess long-term effects. Additionally, the high prevalence of observation techniques (49%) and participant interviews (46.9%) aligns with recommendations favoring qualitative and user-centered evaluation approaches in AR research. However, the limited utilization of validated questionnaires (12.2%) indicates a need for a greater emphasis on standardized measures to ensure the comparability and generalizability of findings across studies, echoing the sentiments of R2. Incorporating more objective assessments (26.9%) can enhance the overall evaluation process by providing a more balanced perspective (R2, R3). Identifying areas for improvement in evaluation methods, the review highlights the relatively low adoption of objective assessment techniques, potentially indicating a bias towards subjective measures (Koumpouros, 2024). Moreover, the infrequent use of control groups in research designs emphasizes the necessity for more rigorous study designs to ascertain the efficacy of AR interventions for individuals with ASD. Aligning with Koumpouros (2024), our study identifies shortcomings in study designs, such as small sample sizes. Looking ahead, researchers should continue exploring innovative evaluation methods and adopting robust study designs to furnish more conclusive evidence regarding the effectiveness of AR interventions for individuals with ASD.

### Looking Forward, Addressing Limitations and Embracing Potential

The scarcity of control groups (2%) highlights the need for more rigorous research designs, as identified in R2, R3. Collaborative efforts from researchers, developers, and practitioners are crucial to design and implement well-controlled studies targeting specific needs and utilizing diverse end-user groups, addressing the limitations identified in R4 (Koumpouros, 2021, 2022). Future research directions should consider ethical considerations and real-world implementation challenges (R5). It is important to note that current AR studies mostly demonstrate associations between AR interventions and positive outcomes, rather than causality. Thus, while promising, these findings should be interpreted with caution. Out of the 49 studies reviewed, 38 reported positive outcomes in terms of improved skills or behaviors, besides general satisfaction and social acceptability. As we look to the future, it is essential to address the limitations identified in the review and embrace the potential of AR interventions for individuals with ASD. The limited representation of certain domains and the lack of standardized evaluation methods present clear areas for improvement in future research. Researchers should prioritize addressing these limitations through more comprehensive study designs and rigorous evaluation protocols. Moreover, efforts should be made to ensure the inclusivity and accessibility of AR interventions for individuals with ASD, taking into account factors such as socioeconomic status and cultural background (Pickard & Ingersoll, 2016). Future AR applications could focus on endorsing social skills in a general population in more complex group settings. By leveraging the engaging and interactive nature of AR technology, developers can create innovative solutions to enhance communication and social interactions among individuals of all ages. There is some evidence that AR interventions are associated with the ability of individuals to generalize social and communication skills learned in AR environments to real-world interactions. Collaborative design between researchers, developers, healthcare professionals, and individuals with ASD and their families will be crucial in driving forward innovation and ensuring the meaningful integration of AR technology into the lives of individuals with ASD. By addressing these limitations and embracing the potential of AR interventions, we can continue to advance our understanding and support of individuals with ASD, ultimately enhancing their quality of life and well-being.

Overall, this review paints an encouraging picture of the potential for AR interventions to address various challenges faced by individuals with ASD. The diverse technological landscape, focus on key areas of need, and emerging evaluation methods point towards promising opportunities for further development and implementation. By addressing the identified limitations and pursuing future research directions outlined in R5, the field of AR interventions has the potential to significantly augment the lives of individuals on the autism spectrum.

## Limitations of the Review

Our systematic review, while comprehensive and informative, is not without limitations. Firstly, we focused solely on English-language publications, potentially excluding valuable research in other languages and introducing language bias. Additionally, the inclusion of conference presentations and lecture notes, while broadening the scope of the review, may have led to variability in the quality of the included studies. Furthermore, the heterogeneity in study designs, intervention strategies, and outcome measures across the reviewed studies complicates the ability to draw definitive conclusions and perform meta-analyses. Another limitation lies in the small sample sizes of many studies and the scarcity of control groups, which restricts the generalizability and reliability of findings. The predominance of subjective evaluation methods, such as participant interviews and observations, over objective measures and validated tools adds to the methodological constraints. Moreover, the underrepresentation of certain domains, such as attention-related issues, rehabilitation training, and daily living skills, highlights gaps in the current literature and the need for more comprehensive exploration of these areas. Lastly, while we employed a rigorous and systematic methodology, the reliance on specific databases may have excluded relevant studies not indexed within these platforms. The findings primarily reflect research involving children and educational settings, with limited focus on diverse subpopulations, such as adults with ASD or individuals with co-occurring conditions. These limitations underscore the importance of addressing these gaps in future research to ensure a more holistic and nuanced understanding of AR interventions for individuals with ASD.

## Conclusion

Technology plays a role, but intervention design, user characteristics, and outcome measures significantly impact results. Variations in sample sizes, outcome measures, intervention durations, and technologies limit direct comparisons and conclusive statements. Choosing the right technology should consider individual needs, learning styles, and accessibility requirements. More research with robust methodologies is needed to establish definitive links between technology and outcomes in ASD interventions. Further exploration is needed in several areas. For example: (i) conduct controlled studies comparing different technologies within specific intervention domains, (ii) investigate the influence of intervention design and user characteristics on outcomes, (iii) explore emerging AR technologies like AR glasses and their potential impact. In conclusion, the review provides a comprehensive overview of the current state of AR interventions for individuals with ASD, highlighting the field’s commitment to leveraging technology to address diverse needs within the ASD community. The review findings suggest that AR interventions can positively impact social interaction skills, communication abilities, and academic performance in individuals with ASD. However, there are significant gaps in the evidence, particularly concerning attention-related issues and emotion recognition. The need for more rigorous and standardized study designs is apparent to better understand and enhance the effectiveness of AR interventions. By addressing these limitations and pursuing future research directions, the field has the potential to significantly augment the lives of individuals on the autism spectrum. Concluding, best practice guidelines for using AR with individuals with ASD include ensuring user-centered design, incorporating feedback from end-users, prioritizing accessibility and usability, and employing rigorous evaluation methods. Practitioners should consider the specific needs and preferences of individuals with ASD when selecting AR interventions.

## Electronic Supplementary Material

Below is the link to the electronic supplementary material.

Figure 2

Figure 3

Table 2

Table 3

Table 4
