# Supplementary figures and images for: Digital Horizons: Enhancing Autism Support with Augmented Reality
Source: J Autism Dev Disord. 2025 Feb 28;56(9):3368–84. doi: 10.1007/s10803-024-06709-4 (PMC13427985; doi:10.1007/s10803-024-06709-4)

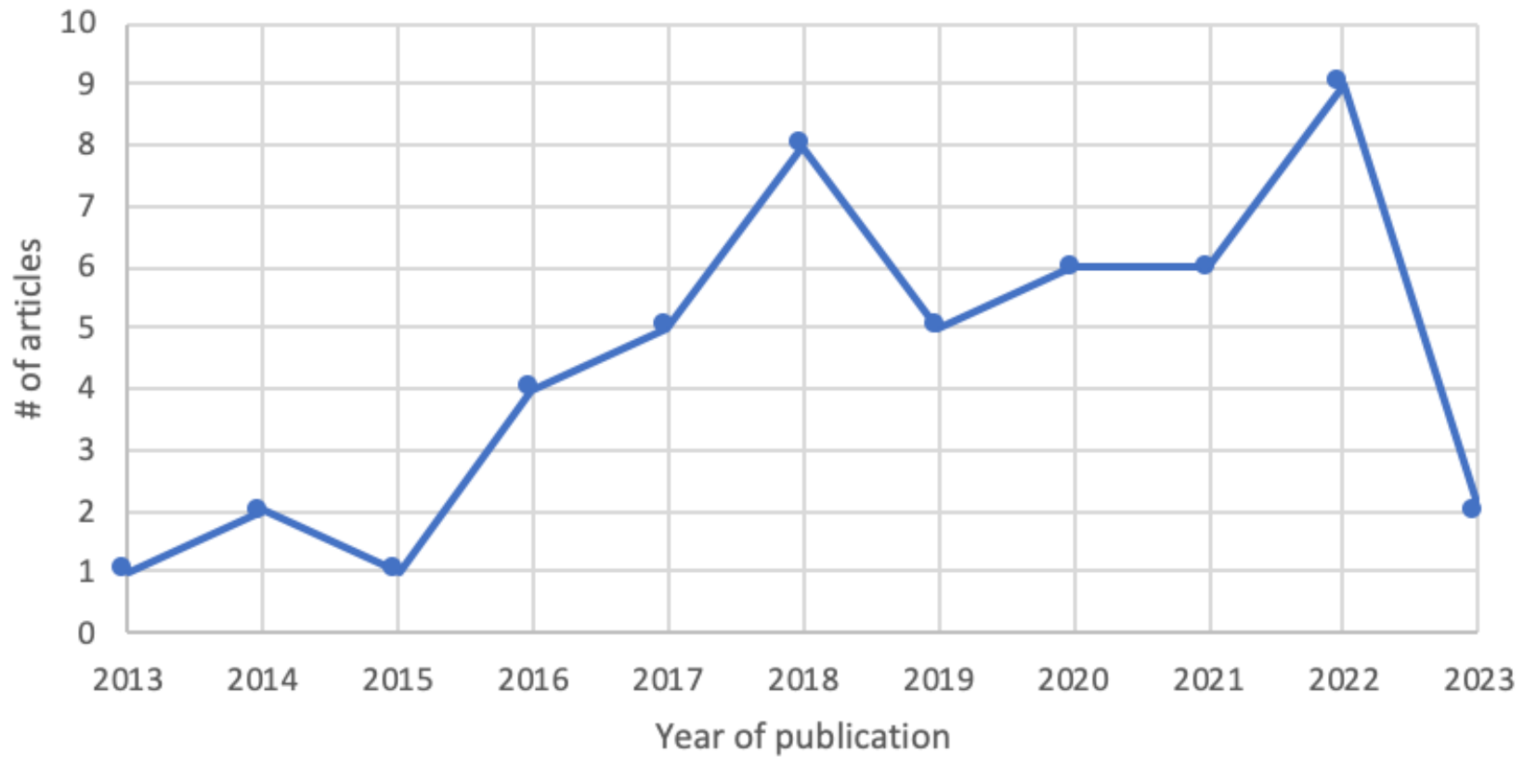

Supplement: Supplementary file 1 — Figure 2 [file 10803_2024_6709_MOESM1_ESM.png]

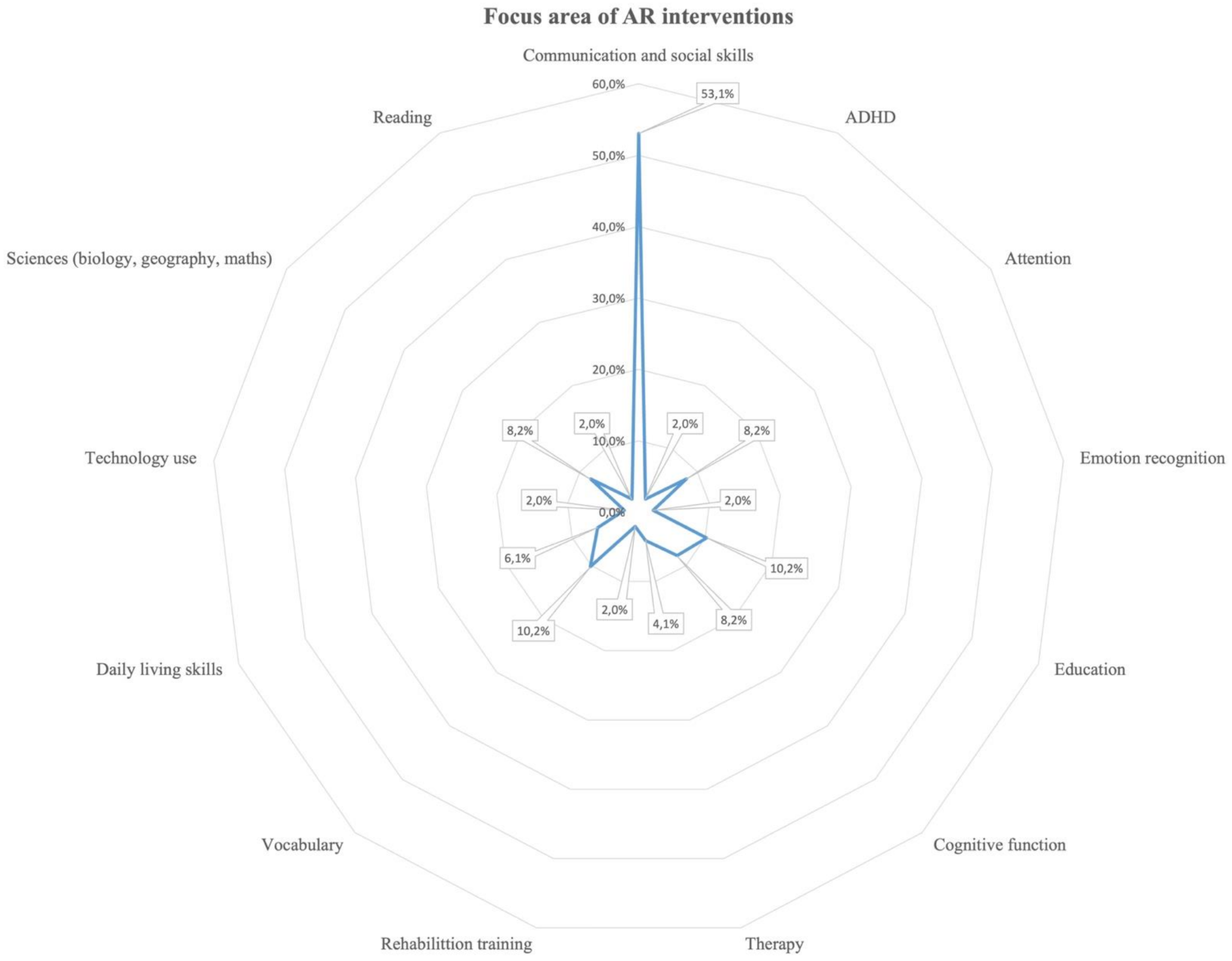

Supplement: Supplementary file 2 — Figure 3 [file 10803_2024_6709_MOESM2_ESM.png]
