# Supplementary material for: Digital Horizons: Enhancing Autism Support with Augmented Reality
Source: J Autism Dev Disord. 2025 Feb 28;56(9):3368–84. doi: 10.1007/s10803-024-06709-4 (PMC13427985; doi:10.1007/s10803-024-06709-4)
Supplement: Supplementary file 3 — Table 2 [file 10803_2024_6709_MOESM3_ESM.docx]

**Table 2.** Scope, Limitations, Future directions

| **#** | **Outcomes** | **Limitations** | **Future plans** |
| --- | --- | --- | --- |
| 1 | Improvements in social communication skills, verbal and nonverbal communication, and repetitive behaviors | Small sample size. Short intervention duration. Lack of control group. Reliance on subjective data without robust reliability measures. | Conduct larger studies with longer interventions and control groups. Investigate the long-term impact of AR interventions. Explore the effectiveness of AR in different settings and for various ASD individuals. |
| 2 | Significant improvements in social awareness, social cognition, social motivation, orientation, memory, attention, and visual perception. The post-intervention survey indicated high satisfaction and usability. | Small ample size. Absensce of a control group. Short-term study. | Use of a randomized control study with a larger sample size to further validate the efficacy of multiplayer game-based dual-task training using AR and PHR for children with autism. |
| 3 | Positive results in terms of reducing irritability, hyperactivity, and social withdrawal in students with ASD. The study demonstrates the feasibility and efficacy of the Empowered Brain intervention in a school setting. | Short duration of the study. Preliminary nature of the evidence. Use of a convenience sample of male participants. | Need for extended longitudinal research to study the longer-term effects of the Empowered Brain intervention, especially considering the typical duration of behavioral interventions in ASD. |
| 4 | Positive outcomes include all eight children finding Glass usable and acceptable. There were no reported negative effects, and caregivers expressed that the experience went better than expected. | Small sample size. Subjective nature of opinions. Need for further research to test acceptability within school environments. | Further research is needed to test the acceptability within school environments. |
| 5 | Improved pointing skills, development of expressive written communication, and engagement in age-appropriate educational content. | The intervention has not yet been implemented. | Implement the remaining requirements, share the prototype with the nonspeaking community, and conduct human subject studies for further refinement. |
| 6 | Positive feedback on the customization options, the freeze feature's utility, and challenges in using picture-based AR in practical therapy settings. | Limited selection of 3D models. Need for further user studies with autistic children to confirm the application's suitability for them. | Development of prototypes with additional features, such as just-in-time content creation and a sequential interface. |
| 7 | The article discusses outcomes related to self-paced learning, listening and understanding instructions, attention span, pronunciation and enunciation improvement, and engagement. Enhanced amount of engagement, focus and imporvement in the learning experience of the english language. | Small sample size. Limitations about the specific characteristics of the participants, and potential biases. | - |
| 8 | Positive effects on improving stereotypic behaviors, compulsiveness, sameness, and restricted behaviors Improved working memory, cognitive flexibility, cognitive inhibition reaction time, and attention with the usage of AR game-based training, compared to the control group that used a tablet app. | Small sample size.Educational and intellectual levels of participants not assessed. Short duration of the training program. | Further research to provide additional evidence concerning the mechanism and evaluate the long-term effect of AR on individuals with ASD. |
| 9 | Improved performance in operation proficiency and duration for six out of ten participants, interest shown by all participants, and improved recognition and imitation abilities for certain expressions. | Influence of lighting and camera factors on video quality. Recognition issues with the DeepLook algorithm for fear expressions. A small group of participants, and the specific age range targeted. | More standardized clinical studies for further verification, including integrating the system on a robot, improving recognition rates, and collaborating with more autism rehabilitation institutions. |
| 10 | The AR system improved facial expression recognition, social interactions, talking, and facial expressions for both autistic and typical children. | Small sample size. | Additional research into the usage of AR technology as a therapeutic device for people with ASD. Larger sample. |
| 11 | Improvement in body language and interaction with teachers. | Lack of these devices in general SE schools. Focus on limited social skills (body language only). Lack of physical contact. Selection of high functional ASD children only. | Include low and middle functional ASD children in future experiments. |
| 12 | Improved desire to communicate. Increased accuracy rate of help requests. Enhanced communication level. | Small sample size of participants.Challenges in finding a large number of suitable ASD participants. Difficulty in achieving normal distribution. | Conduct more high-quality studies. Add modules about life scenarios and not only request-assistance. |
| 13 | Improved shopping skills for individuals with ASD, enhanced accessibility, and user-friendly features. | Small sample size. Challenges in collecting a diverse set of images for the fruits and vegetables recognition model. | Planning a formal user study after the reopening of businesses in New York City, with IRB approval ready for tests with ASD users. Adding more images and categories to the image selection feature and expanding the fruits and vegetables recognition model. |
| 14 | - | Challenges in concealing real-world objects due to the nature of OST AR displays, potential misalignment between the rendered model and the subject’s head based on different pupil distances. | Integration of facial expression extraction process. Conduct studies on the influence of rendered emojis on users' perception of emotions. Explore the impact of mismatched emoji expressions on viewers' perception. |
| 15 | Positive feedback from both adults and children in terms of usability and interest. Satisfying results in terms of accuracy during pilot studies. Identified limitations in the performance of the object recognition module. | Accuracy issues in the object recognition module, particularly with the expanding vocabularies of children. Limited time for each child during pilot studies. | Integration of a reinforcement learning module to improve the accuracy of the object recognition algorithm. |
| 16 | Improved social interaction skills in terms of greeting behavior. Positive changes in the participants' behavior were observed. | Short-term study. Small sample size. Challenges in determining actual improvement in complex reciprocal social behaviors. Focus specifically on greeting behavior. | Recruiting more participants, analyzing individual differences (e.g., eye contact and facial expressions), and long-term observation studies. |
| 17 | Reduction in ADHD-related symptoms (hyperactivity, inattention, impulsivity) in participants with ASD after using the Empowered Brain system. | No control group. Small sample size. Potential expectancy effect. Lack of a broader generalizability due to the limited sample size and absence of a control group. | Further research is required to understand the clinical importance of observed changes. Longitudinal studies with control groups and larger sample sizes. |
| 18 | Positive perceptions of the smartglasses intervention by educators, improvements in the student's social and academic skills as reported by educators, and overall feasibility and practicality of delivering the intervention in a classroom. | Lack of a control group. Small sample size. Absence of qualitative approaches. | Future research with a larger sample size and broader generalizability. |
| 19 | Improved learning of autistic children, positive impact observed during testing. | Fearfulness observed in some participants due to loud audio or display of frightful objects. Small sample size. Short-term study. | - |
| 20 | Introducing AR technologies to conventional social stories, understanding user behaviors, and providing a framework for future research. | The system is in its initial phase and has not been tested on people. | A series of usability study will be conducted to validate the design and to get more information to improve and refine the system. Using participants' faces to create 3D virtual characters, preparing various backgrounds for different social situations, and incorporating new mechanisms to make the system more game-like. |
| 21 | Improvement in social communication, social cognition, social motivation. | Single-case study design. Limited generalizability. Lack of daily/near daily data points. | Broader testing and replication of results. Potential adjustments for different educational systems and cultures. |
| 22 | No statistically significant differences found in the development of skills between AR and non-AR intervention groups. | Gender imbalance in sample. Small sample size. Lack of random assignment to experimental and control groups. Potential bias from professionals conducting intervention and assessment. Test sensitivity concerns. | Conducting the study with a larger sample. Exploring other AR applications and activities. Extending intervention duration. Investigating effects on older children with ASD. |
| 23 | Improvement in learning and understanding teaching content, significant improvement in learning effectiveness, mastery of abstract social concepts and structure of complex social relationships. | Small sample size. | Improve ARCM social-training system based on collected first-hand information. |
| 24 | Significant improvement in communication ability. | Small sample size. Non generalizable results. | - |
| 25 | Encouraging results that showed a possible way of understanding ASD children better. | Difficulty in judging the attention level of the ASD children. | Continuous iterative learning to improve the system's effectiveness in avoiding children's physical exhaustion. |
| 26 | Improvements in social skills, attention, and engagement among autistic children, as observed through the training sessions and evaluations. | Small sample size. Potential biases in assessment, and generalizability of findings. | Adding different theater-based story scripts to further improve the training system and gather more experimental data for future studies. |
| 27 | The smartglasses system (Brain Power Autism System) was well tolerated and usable by a diverse range of individuals with ASD. The majority of users demonstrated tolerability on all measures, and caregivers reported positive responses to the smartglasses experience. | Moderate sample size. Customized nature of the smartglasses system (BPAS) which may limit generalizability to other smartglasses or software apps. | Further research to focus on improving smartglasses design and exploring their efficacy in helping with social communication in children and adults with ASD. |
| 28 | Improvement in emotional recognition and social skills. | Small sample size and only included participants without intellectual disabilities, limiting the generalizability of the findings. No control group. | Conducting further research with larger sample sizes and including participants with intellectual disabilities. Exploring AR technology in other areas of ASD intervention. |
| 29 | Improvement in RJA (Response to Joint Attention) skills. Generalization of skills to novel situations. Maintenance of skills observed one month after the end of the intervention. | ESCS is designed for younger children. Lack of standardized JA assessment tools for older children. No systematic evaluation of procedural fidelity. Small sample size and specific context of study. Need for further studies in different contexts with more participants. | Exploring the impact of ADHD symptoms on learning outcomes in AR or VR-TMI, using different hardware and visual cues. Exploring effects of more comprehensive interventions. Tracking gaze using eye-tracking systems. New version of Pictogram Room with updated games and internal data capturing. |
| 30 | Improved the appropriate recognition and response to facial emotional expressions seen in the situational task. | Small sample size. | Involve more participants with ASD. Investigate AR more thoroughly. |
| 31 | Increased task completion rates and improved concentration, willingness to participate, and frequency of eye contact among the children. | Small sample size. | Cancel the screen obstruction in communication and consider tangible masks for face-to-face communication in the future. |
| 32 | - | Technical difficulties such as image processing and pattern recognition, dependency on the system for interaction. The intervention has not yet been implemented. | Include face recognition in the background, and use AI to mirror human expressions. |
| 33 | AR instructional intervention was effective in improving science vocabulary acquisition for all participants. | Small sample size. Lack of maintenance probes. Need for further examination of long-term effects and comparison with other instructional strategies. Only 1 person with ASD. | Replicate the methods, include other disability populations and age groups, explore AR instruction in other content areas, and investigate various instructional AR components systematically. |
| 34 | Satisfactory performance of the proposed AR/VR system in laboratory conditions and positive feedback from psychologists and teachers. | - | Further evaluation of the system with ASD children and collecting feedback from parents, teachers, and psychologists. |
| 35 | Reduction in teachers' burden and workload, increased multitasking ability, and positive feedback from teachers about the ease of use and usefulness of MOBIS. | - | Analyze how MOBIS supports skills generalization and evaluate the impact of an "appropriate AR visor" on the prompts given by teachers to children with autism. |
| 36 | The AR features implemented were appreciated by parents saying that album creator application with the sound and music would be an excellent memoire creation tool. | Support for only specific types of 3D models, the inability for children to pause audio or video during playback, and the lack of options for instructors to upload custom 3D images. | Implementing features for progress tracking and evaluation, addressing limitations, and enhancing the application based on feedback. |
| 37 | The article discusses the outcomes of the design process, including the successful involvement of stakeholders (children, parents, specialists, and caregivers) in the co-design process and the positive impact on the quality and accessibility of the Yohka application. | - | Durther evaluation and testing of the design process and its effect on the overall user experience. |
| 38 | Marker-based AR showed usability challenges for the ASD people. | Usability challenges with the AR-based mode, reliance on instructors for individuals with autism, and the need for further exploration of alternative AR-based solutions. | Exploring brain-sensing technologies, developing adaptive intelligent tutoring systems, comparing the AR-based tool with other advanced AR devices, and exploring alternative AR-based solutions for individuals with autism. |
| 39 | The AR courses were more useful than static graphic courses. Improved academic performance for ASD students using BLS (Augmented Reality Animation), positive feedback from Special Education Teachers on the usability of the e-courseware, and insights into the effectiveness of augmented reality in assisting ASD students. | - | Need for better implementation strategies to address the challenges of providing costly Augmented Reality Animation e-courseware in all classes. The study highlights the importance of training SpeEdu teachers in using such technology in classrooms. |
| 40 | - | Not yet completed or implemented. | Ethics approval for a co-design process with ASD kids and investigate some research issues. |
| 41 | The results from the practitioners confirmed the application's well-being potential and acceptability and helped gain insight about its usability. | The only feedback was from practitioners and not ASD children. | Evaluate the application with ASD children as participants. |
| 42 | Positive feedback on the prototype, satisfaction with cognitive games, recommendation for the mobile application. | Complexity of the hybrid methodology, time-consuming nature of the 10 phases. | Use of Tinkercad with Arduino for optimization, Unity3D for application operation, Vuforia SDK for augmented reality in other systems. Improve the app's optimization and operation. |
| 43 | MARVoc V1 and MARVoc V2 prototypes, with enhancements such as a robotic avatar, interactive dashboard, educational games, and improved user interface. Positive feedback and recommendations from experts. | Challenges with scanning cards for children with motor limitations. Specific limitations of the study were not explicitly mentioned. | Conducting a single-subject design study with children with ASD, parents, and teachers to evaluate the effectiveness of the intervention. |
| 44 | Preliminary findings indicate effectiveness in usability and engagement. | Experimental process is in its early stages. Needs further testing and refinement. | Explore effectiveness with a larger sample size, collect feedback from parents, teachers, and psychologists. Integrate more daily life skills in the app. Test the system on ASD children. |
| 45 | Positive experiences with AR applications among both professors and students. AR applications were found to enhance learning, improve engagement, and reduce stress. Collaborative AR games were particularly effective in fostering teamwork and communication skills. | Minor performance and stability issues with the devices, especially during fast movements or when multiple touch screen inputs occurred simultaneously. | Introducing a component based on Amazon's Alexa for evaluating students using AR apps, utilizing voice recognition for answers and automatic evaluation. |
| 46 | Promising results in feasibility and usability, satisfying engagement of children and parents. | Limited training sets affecting accuracy, lack of conclusive findings in clinical aspects, limited exploration of offline deep-learning-based object recognition. | Further exploration of offline deep-learning-based object recognition, improvement of accuracy, integration with classroom-based intervention. |
| 47 | - | Not yet implemented. | Perform a case study to observe the impact of the game on children's interaction, assess usability, and evaluate its effects on emotion reactions. |
| 48 | High engagement, Improved social behaviors and facial expression recognition. | Small sample size. Lack of automated agent response generation for children with phonation disorders. | Further development of FaceMe system. Assessment of typically developed children. Expansion of interaction scenarios. Enlist additional participants. Assess the performance of subjects in both short-term and extended usage scenarios. |
| 49 | Identified visual processing differences and preferences in children with autism, suggested recommendations for improving user interface design for accessibility. Images enhance ASD children's comprehension of task, size and placement of icons incluenced the information processing, intricate interfaces are not helpful. | Experimental setup using screenshots on a monitor rather than actual mobile devices. Potential limitations in the generalizability of findings due to the specific app and context. Setup problems: eye-tracking technology problems due to device (mobile). | Future studies to evaluate more UI pages with varying complexity and restore natural interaction environment. |
