# Supplementary material for: Digital Horizons: Enhancing Autism Support with Augmented Reality
Source: J Autism Dev Disord. 2025 Feb 28;56(9):3368–84. doi: 10.1007/s10803-024-06709-4 (PMC13427985; doi:10.1007/s10803-024-06709-4)
Supplement: Supplementary file 4 — Table 3 [file 10803_2024_6709_MOESM4_ESM.docx]

**Table 3.** Taxonomy-based results according to Koumpouros (2024)

| **#** | **Objective of the Study** | | | **Educational Purpose** | | | **Research Focus** | | | | | |
| --- | --- | --- | --- | --- | --- | --- | --- | --- | --- | --- | --- | --- |
|  | **Enhancing learning** | **Pedagogical approaches** | **Technical Aspects** | **Training** | **Teaching** | **Observing** | **Pedagogical Integration** | **Technical Development** | **Learning Outcomes** | **Motivation and Engagement** | **Accessibility and Inclusivity** | **Teacher Training and Professional Development** |
| 1 |  |  | x | x |  |  |  | x | x | x | x |  |
| 2 |  | x | x | x |  |  |  | x | x | x | x |  |
| 3 | x |  |  |  | x |  | x |  | x | x | x | x |
| 4 |  |  | x |  | x |  | x |  | x |  | x |  |
| 5 |  |  | x |  | x | x | x | x | x | x | x | x |
| 6 |  |  | x | x | x |  | x | x | x |  |  |  |
| 7 |  | x | x |  | x | x | x | x | x | x |  |  |
| 8 | x | x | x |  | x |  | x | x | x | x |  |  |
| 9 |  |  | x | x |  |  |  | x | x |  |  |  |
| 10 |  | x | x | x |  |  |  | x | x |  |  |  |
| 11 |  | x | x | x |  | x | x | x | x | x |  |  |
| 12 | x | x | x | x | x |  | x | x | x | x | x | x |
| 13 |  | x | x | x |  |  | x | x | x |  | x |  |
| 14 |  |  | x |  |  | x |  | x |  |  | x |  |
| 15 |  | x | x | x | x | x | x | x | x | x | x |  |
| 16 | x | x | x | x | x |  | x | x | x | x |  |  |
| 17 | x | x | x | x | x | x | x | x | x | x | x | x |
| 18 |  | x | x |  | x |  | x | x | x | x | x | x |
| 19 |  | x | x |  | x | x | x | x | x | x | x | x |
| 20 | x | x | x | x | x | x | x | x | x | x | x | x |
| 21 |  |  | x |  | x |  | x | x | x | x | x |  |
| 22 |  | x | x |  | x | x | x |  | x | x |  |  |
| 23 | x | x |  | x | x | x | x | x | x | x |  |  |
| 24 | x | x | x | x | x | x | x | x | x | x |  |  |
| 25 | x |  | x | x |  |  | x | x |  | x |  |  |
| 26 |  | x |  | x |  |  | x |  | x | x |  |  |
| 27 | x | x | x | x | x |  | x | x | x | x |  |  |
| 28 |  | x | x | x |  |  | x | x | x | x |  |  |
| 29 |  | x | x |  | x | x | x |  | x | x | x |  |
| 30 | x | x |  | x |  |  | x |  | x | x |  |  |
| 31 | x | x | x | x |  |  | x | x | x | x |  |  |
| 32 |  | x | x | x |  |  | x | x |  |  | x |  |
| 33 |  | x | x | x | x |  | x |  | x | x | x |  |
| 34 | x |  | x | x |  |  | x | x | x |  |  |  |
| 35 | x | x | x | x |  | x | x |  |  | x |  | x |
| 36 | x | x | x | x | x |  | x | x |  |  | x | x |
| 37 | x | x | x | x | x | x | x | x | x |  | x |  |
| 38 | x | x |  | x | x | x | x | x | x | x | x |  |
| 39 | x | x |  | x | x |  | x | x | x | x |  | x |
| 40 | x |  | x | x | x |  | x |  | x | x |  |  |
| 41 |  | x |  | x |  | x | x |  |  | x | x | x |
| 42 | x |  | x | x | x |  |  | x | x |  | x |  |
| 43 | x |  | x | x | x |  | x | x | x |  | x |  |
| 44 | x | x |  | x | x |  | x | x | x | x | x |  |
| 45 | x | x | x |  | x | x | x | x | x | x |  | x |
| 46 | x |  | x |  | x |  | x | x |  |  |  |  |
| 47 | x |  |  | x |  |  | x |  | x | x |  |  |
| 48 |  | x | x | x |  | x | x | x | x | x |  |  |
| 49 |  |  | x |  |  | x |  |  |  |  | x |  |
