# Supplementary material for: Digital Horizons: Enhancing Autism Support with Augmented Reality
Source: J Autism Dev Disord. 2025 Feb 28;56(9):3368–84. doi: 10.1007/s10803-024-06709-4 (PMC13427985; doi:10.1007/s10803-024-06709-4)
Supplement: Supplementary file 5 — Table 4 [file 10803_2024_6709_MOESM5_ESM.docx]

**Table 4.** Summary of Key Findings

| **#** | **Device Used** | **AR Type** | **Key Outcomes** | **Limitations** |
| --- | --- | --- | --- | --- |
| 1 | Google Glass Explorer Edition v2 | M | Improvements in social communication skills, verbal and nonverbal communication, and repetitive behaviors. | Small sample size. Short intervention duration. Lack of control group. Subjective data without robust measures. |
| 2 | Kinect | S | Significant improvements in social awareness, social cognition, social motivation, orientation, memory, attention, and visual perception. High satisfaction and usability. | Small sample size. Absence of a control group. Short-term study. |
| 3 | Empowered Brain Smartglasses | S | Positive results in reducing irritability, hyperactivity, and social withdrawal in students with ASD. Demonstrated feasibility and efficacy in school settings. | Short study duration. Preliminary evidence. Convenience sample of male participants. |
| 4 | Glass Enterprise Edition | S | All eight children found Glass usable and acceptable. No negative effects reported, caregivers expressed satisfaction. | Small sample size. Subjective opinions. Need for further research in school environments. |
| 5 | Hololens 2 | S | Improved pointing skills, expressive written communication, and engagement in educational content. | The intervention has not yet been implemented. |
| 6 | Smartphone | M | Positive feedback on customization options and the freeze feature. Addressed challenges in using picture-based AR in therapy. | Limited selection of 3D models. Need for further user studies with autistic children. |
| 7 | Smartphone | M | Enhanced engagement, focus, and learning experience in English vocabulary. Improved attention span, listening, and instruction-following skills. | Small sample size. Potential participant biases. |
| 8 | UINCARE-82B (PC, Kinect), CoTras Device | M | Positive effects on stereotypic behaviors, compulsiveness, and restricted behaviors. Improved working memory, cognitive flexibility, and attention. | Small sample size. Educational and intellectual levels not assessed. Short training program duration. |
| 9 | PAD with training system, PC with analysis system, mini Bluetooth speaker, D435 camera | S | Improved operation proficiency and recognition/imitation abilities for certain expressions. Interest shown by all participants. | Influence of lighting and camera factors. Recognition issues with fear expressions. Small group size. |
| 10 | Kinect | S | Improved facial expression recognition, social interactions, and facial expressions in autistic and typical children. | Small sample size. |
| 11 | Kinect | S | Improved body language and interaction with teachers. | Lack of devices in schools. Focus on limited social skills (body language only). Selection bias. |
| 12 | Tablet | M | Improved communication desire, help-request accuracy, and enhanced communication levels. | Small sample size. Challenges in finding suitable ASD participants. |
| 13 | Smartphone | M | Improved shopping skills for individuals with ASD, enhanced accessibility, and user-friendly features. | Small sample size. Challenges in collecting diverse images for the recognition model. |
| 14 | Magic Leap One Headset | M | Positive feedback from both adults and children. Satisfying results in terms of usability and accuracy during pilot studies. | Accuracy issues in object recognition. Limited time for each child during pilot studies. |
| 15 | Smartphone | M | Positive feedback and significant results in word-learning app usability for children with autism. | Limited time per child in pilot tests. Object recognition limitations. |
| 16 | Tablet | M | Improved social interaction skills in terms of greeting behavior. Positive changes in participants' behavior. | Short-term study. Small sample size. Focus specifically on greeting behavior. |
| 17 | Empowered Brain Smartglasses | S | Reduction in ADHD-related symptoms (hyperactivity, inattention, impulsivity) in participants with ASD. | No control group. Small sample size. Potential expectancy effect. |
| 18 | Empowered Brain Smartglasses | S | Positive perceptions by educators. Improvements in students' social and academic skills. Overall feasibility in classroom settings. | Lack of a control group. Small sample size. Absence of qualitative approaches. |
| 19 | Kinect, Projector, Webcam | M, P | Improvements in social skills, attention, and engagement among autistic children through AR-based interventions. | Small sample size. Potential biases in assessment. |
| 20 | Laptop | M | Enhanced social communication through augmented reality-based social stories training systems. | Lack of a control group. Small sample size. No maintenance probes. |
| 21 | Empowered Brain Smartglasses | S | Improved social communication, cognition, and motivation via educator and parent assessments. | Preliminary study. Small sample size. No control group. |
| 22 | Smartphone | S | Improvement in social communication and social cognition skills for children with ASD. | Small sample size. Limited generalizability due to single-case design. |
| 23 | Laptop | M | Improvement in learning effectiveness, mastery of abstract social concepts, and understanding of complex social relationships. | Small sample size. No systematic evaluation of procedural fidelity. |
| 24 | Smartphone, Tablet | M | Significant improvement in communication ability using AR-based PECS methods. | Small sample size. Non-generalizable results. |
| 25 | EEG Helmet, Laptop | M | Encouraging results in understanding ASD children better. Neural activity patterns were identified, aiding personalized interventions. | Difficulty in judging the attention level of ASD children. |
| 26 | Tablet | M | Improvements in symbolic play and social skills for autistic children through AR and theater-based games. | Small sample size. Potential biases in assessment. |
| 27 | Google Glass Explorer Edition and Enterprise Edition | S | High tolerability and usability for a diverse sample. Positive caregiver feedback. | Moderate sample size. Customized nature of the system may limit generalizability. |
| 28 | Tablet PC | O | Improvement in emotional recognition and social skills for adolescents with ASD. | Small sample size. Participants without intellectual disabilities only. |
| 29 | Kinect, Projector, Interactive Digital Whiteboard | S | Improvement in RJA (Response to Joint Attention) skills. Maintenance of skills one month after intervention. | Lack of standardized assessment tools for older children. Small sample size. |
| 30 | PC, Webcam | S | Improved recognition and response to facial emotional expressions in situational tasks. | Small sample size. |
| 31 | Laptop, Monitor | S | Enhanced communication through cartoon-masked chat systems for ASD children. | Small sample size. Dependency on technology for communication. |
| 32 | PC, Monitor | S | Improved communication and engagement through face morphing and substitution techniques. | Technical challenges in image processing and pattern recognition. |
| 33 | iPad | M | AR instructional intervention effectively improved science vocabulary acquisition. | Small sample size. Lack of maintenance probes. |
| 34 | Projector, Camera | P | Satisfactory performance of AR/VR system for training ASD children. | No specific limitations mentioned. |
| 35 | Smartphone | P | Reduced teachers' burden and increased multitasking ability with AR applications. | Limited testing with large sample sizes. |
| 36 | Tablet, Smartphone | M | Enhanced learning experiences for children with autism through AR-assisted learning. | Limited generalizability due to small sample size. |
| 37 | Smartphone | M | Co-design process led to positive outcomes in AR storybook development for ASD children. | Challenges in usability and user interface design. |
| 38 | PC | M | Improved procedural task training outcomes through AR applications. | Usability challenges for AR-based modes and reliance on instructors. |
| 39 | Tablet, Smartphone | M | Positive impact of AR e-courseware on academic performance and skill acquisition. | High costs and need for teacher training in using AR technology. |
| 40 | Smartphone | S | Promoted social skills through AR-based face mask applications for ASD children. | Intervention not yet completed or implemented. |
| 41 | HTC Vive Pro, Zed Mini Camera | S | Promising results in feasibility, usability, and engagement of children and parents with AR apps. | Limited training datasets affecting recognition accuracy and need for classroom-based testing. |
| 42 | Smartphone | M | Positive feedback on prototype, cognitive games, and recommendations for mobile applications. | Complexity of hybrid methodology and time-consuming 10-phase process. |
| 43 | Smartphone, Tablet | M | Promising results in mixed-reality learning platforms for ASD children. | Usability testing still ongoing. |
| 44 | Smartphone | M | Enhanced daily life skills learning for ASD children through AR apps. | Needs more studies with larger sample sizes. |
| 45 | Smartphone, Tablet | M | Enhanced learning and usability for ASD children through AR applications. | Limited studies on long-term usability and real-world classroom applications. |
| 46 | Smartphone | S | Effective vocabulary learning through lightweight AR applications. | Small sample size and need for better object recognition algorithms. |
| 47 | Smartphone, Tablet, Laptop | M | Improved cognitive and social skills through AR-based interventions for ASD children. | Intervention yet to be implemented; limited usability testing. |
| 48 | Laptop | S | High engagement, improved social behaviors, and facial expression recognition in ASD children. | Small sample size and lack of automated response generation for phonation disorders. |
| 49 | Smartphone | M | Identified visual processing differences and improved UI design recommendations for ASD users. | Eye-tracking technology issues and setup constraints limited data collection and generalizability. |
